# Rapid regulation of endoplasmic reticulum dynamics in dendritic spines by NMDA receptor activation

**DOI:** 10.1186/s13041-014-0060-3

**Published:** 2014-08-19

**Authors:** Ai Na Ng, Andrew J Doherty, Paul J Lombroso, Nigel J Emptage, Graham L Collingridge

**Affiliations:** 1 Centre for Synaptic Plasticity, School of Physiology and Pharmacology, University of Bristol, Dorothy Hodgkin Building, Bristol BS1 3NY, UK; 2 Child Study Centre and Departments of Neurobiology and Psychiatry, Yale University, New Haven 06520, CT, USA; 3 Department of Pharmacology, University of Oxford, Oxford OX1 3QT, UK

**Keywords:** NMDA, Tyrosine phosphatase STEP, Endoplasmic reticulum, Dendritic spine, Hippocampus, Live cell imaging, Primary culture

## Abstract

Endoplasmic reticulum (ER) is motile within dendritic spines, but the mechanisms underlying its regulation are poorly understood. To address this issue, we have simultaneously imaged morphology and ER content of dendritic spines in cultured dissociated mouse hippocampal neurons. Over a 10 min period, spines were highly dynamic, with spines both increasing and decreasing in volume. ER was present in approximately 50% of spines and was also highly dynamic, with a net increase over this period of time. Inhibition of the endogenous activation of NMDA receptors resulted in a reduction in ER growth. Conversely, augmentation of the synaptic activation of NMDA receptors, by elimination of striatal-enriched protein tyrosine phosphatase (STEP), resulted in enhanced ER growth. Therefore, NMDA receptors rapidly regulate spine ER dynamics.

## Background

Endoplasmic reticulum (ER) is present in hippocampal dendritic spines [1]–[6] where it may serve multiple functions including a role in synaptic plasticity [1]. The use of the fluorescent-tagged ER label, which specifically distributes with ER, has revealed that spine ER can be highly dynamic [2],[3],[5]. However, the mechanisms that regulate spine ER content and dynamics are poorly understood.

In hippocampal neurons, activation of NMDA receptors promotes dynamic changes in spine morphology [7]–[12]. The tyrosine phosphatase STEP is an endogenous negative regulator of NMDA receptors that dephosphorylates tyrosine (Y^1472^) on GluN2B, leading to internalization of GluN1/GluN2B NMDA receptors [13]–[19]. In the present study, we investigated the role of NMDA receptors, and their regulation by STEP, in the modulation of the dynamics and ER content of spines. We found that activation of NMDA receptors drives rapid increases and decreases in spine volume and associated ER content. We also found that STEP provided a powerful inhibition of this process, a regulation that occurs primarily in spines containing ER. These data suggest that NMDA receptors, STEP and ER are intricately linked in the rapid regulation of spine growth.

## Results

### Rapid alterations in spine morphology of cultured hippocampal neurons

**Figure 1:**
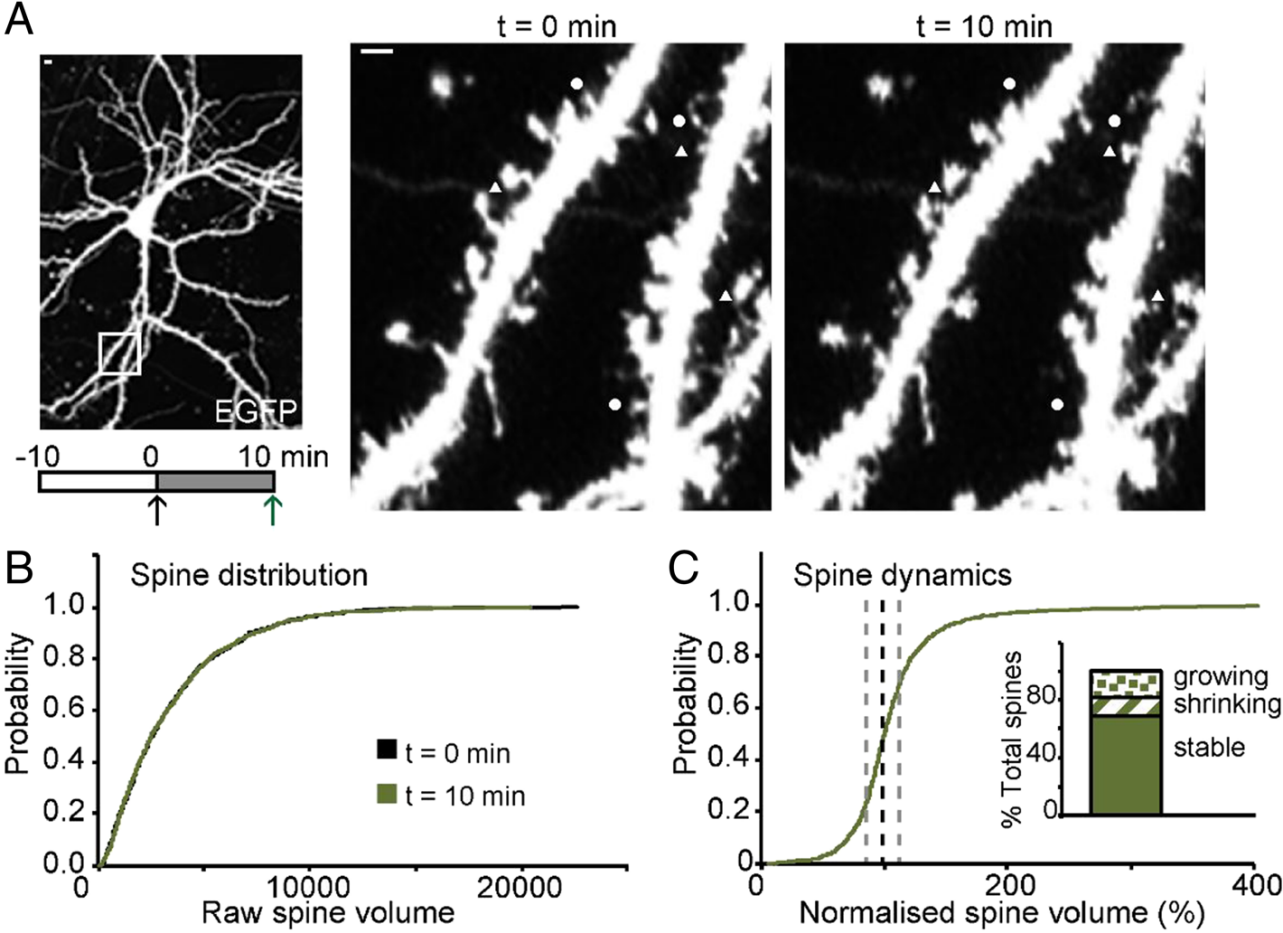
**Spontaneous morphological changes of hippocampal dendritic spines under basal conditions. (A)** Representative images of a cultured hippocampal neuron transfected to express cytosolic EGFP. Zoomed images of the same dendrite at two time points, as shown by the schematic diagram below. Examples of growing spines and shrinking spines are depicted by triangles and circles, respectively. Scale bar in all panels: 5 μm. **(B)** A cumulative probability plot of spine volume (arbitrary units) for 1421 spines from 20 neurons at t = 0 (black) and t = 10 (green). **(C)** A cumulative probability plot of normalised spine volume to show spine dynamics over a 10 min period. To obtain this, the volume at t = 10 was divided by the volume at t = 0 for each of the 1421 spines. The dotted lines depict no change (black) or a change of ± 25% (grey). Inset: Data were subdivided into growing, shrinking or “stable” spines, based on the changes (±25%) over this 10 min period, for illustrative purposes.

To estimate spine dynamics we used cytosolic EGFP. We measured the size of dendritic spines at two time points, commencing 10 min after the neurons had been placed in the imaging medium (t = 0) and 10 min later (t = 10). By measuring how spines altered over this 10 min interval we could estimate the extent of spine dynamics (Figure 1A inset).

Under basal conditions, spines were highly unstable structures, with some spines appearing, some disappearing and most altering in size and shape (Figure 1A). To quantify this effect we measured the volume of 1,421 spines from 20 neurons. Surprisingly, there was little change in the overall distribution of spine volumes, as clearly illustrated by the overlapping cumulative distribution plots of spine volume (Figure 1B). Next, we divided the volume at t = 10 by that at t = 0 for each spine and constructed cumulative distribution plots of the normalised data, as an estimate of spine dynamics (Figure 1C). This demonstrates that the volume of individual spines over time is highly variable. There was no correlation between spine dynamics and initial spine volume (data not shown). To obtain a semi-quantitative estimate of the extent of spine dynamics we subdivided spines into three groups, based on the change in individual spine volume: growing spines (>25% increase), shrinking spines (>25% decrease) and “stable spines” (25% or less change in spine volume). Using these criteria, 68% of spines were “stable,” 19% were growing and 13% were shrinking (Figure 1C inset).

### Spontaneous ER dynamics in hippocampal dendritic spines

**Figure 2:**
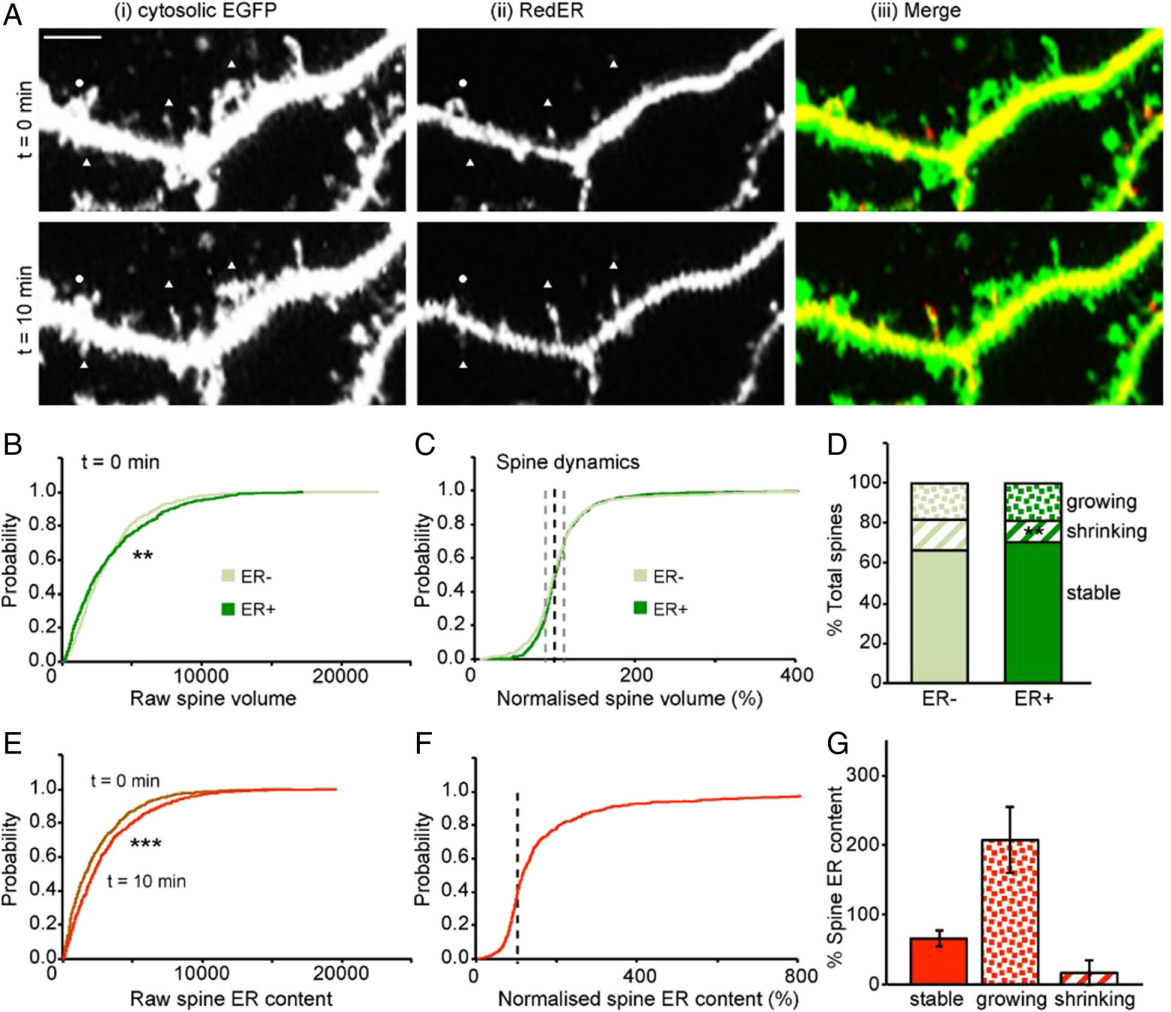
**Spontaneous ER dynamics in dendritic spines. (A)** Representative images of the same dendrite at two time points to show dynamic events at, (i) the level of spine volume and (ii) ER content of spines. Examples of expanding ER and shrinking ER are depicted by triangles and circles, respectively. Scale bar (applies to all panels): 5 μm. **(B)** Cumulative probability plot to compare spine distribution of ER + and ER- spines at t = 0 (K-S test, P < 0.01). **(C)** Cumulative probability plot of normalised spine volumes to compare the dynamics of ER + and ER- spines over a 10 min period. **(D)** A comparison of the proportion of growing, “stable” and shrinking ER + and ER- spines (Z test, P < 0.01 for shrinking). **(E)** Cumulative probability plot of ER content of spines at t = 0 and t = 10 min (K-S test, P < 0.001). **(F)** Cumulative probability plot of normalised ER to illustrate its dynamics over a 10 min period. **(G)** Changes in ER content for the three spine subgroups.

To be able to compare ER and spine dynamics, we simultaneously expressed cytosolic EGFP (Figure 2Ai) and RedER (Figure 2Aii). Approximately 50% (743 of the 1,421) of spines analyzed contained ER at either t = 0 or t = 10 min, and were defined as ER positive (ER+). The remainder did not express detectable ER at either of these time-points and so were defined as ER negative (ER-).

The distribution of spine volume between the ER + and ER- spines was similar (Figure 2B). Within the ER + population, there was a positive correlation between spine volume and ER content (Spearman’s correlation coefficient of 0.492). In terms of spine dynamics (Figure 2C) there were fewer shrinking spines and correspondingly more “stable” spines than in the ER- group (Figure 2D). The effects on spine volume, although small, were however associated with more pronounced effects on ER itself. Thus, there was a substantial increase in ER content in spines on average (Figure 2E). Analysis of the changes in individual spines revealed that this was due to an increase in ER content in the majority of spines, although some spines had a reduction in their ER content (Figure 2F). When the change in ER spine content was compared for growing, shrinking and “stable” spines (Figure 2G) or the entire ER + spine population, it was evident that the largest changes were associated with the growing spines.

These results suggest that there are two modes of dynamic events in operation under basal conditions, one at the level of spine volume and the other at the level of ER content in spines.

### NMDA receptors activation promotes spine and ER dynamics

The extent of the dynamics of both spine morphology and ER, over the 10 min observation period, was surprising. Since NMDA receptor activation has been shown to affect spine dynamics [7]–[12], we wondered whether the dynamic changes were driven by activation of NMDA receptors, generated by spontaneous synaptic activity within the cultures. To test for this we included a specific NMDA receptor antagonist in the medium and measured the spine and ER parameters at the equivalent two time points. We used L689,560, a high affinity, competitive antagonist at the glycine site of the NMDA receptor [20] that we have used extensively previously [21]–[23]. We selected this compound since it is independent of L-glutamate concentration, which may vary considerably within a dissociated culture preparation. We used a relatively high concentration (5 uM, which is approximately 40x the K_b_ of 130 nM obtained in a cortical slice preparation, which has a near saturating glycine concentration [20]) to compensate for possible accumulation of glycine or D-serine in the cultures. We analyzed a total of 1,383 spines from 21 neurons treated with L689,560. Interleaved vehicle experiments are included within the data presented in Figure 2.

**Figure 3:**
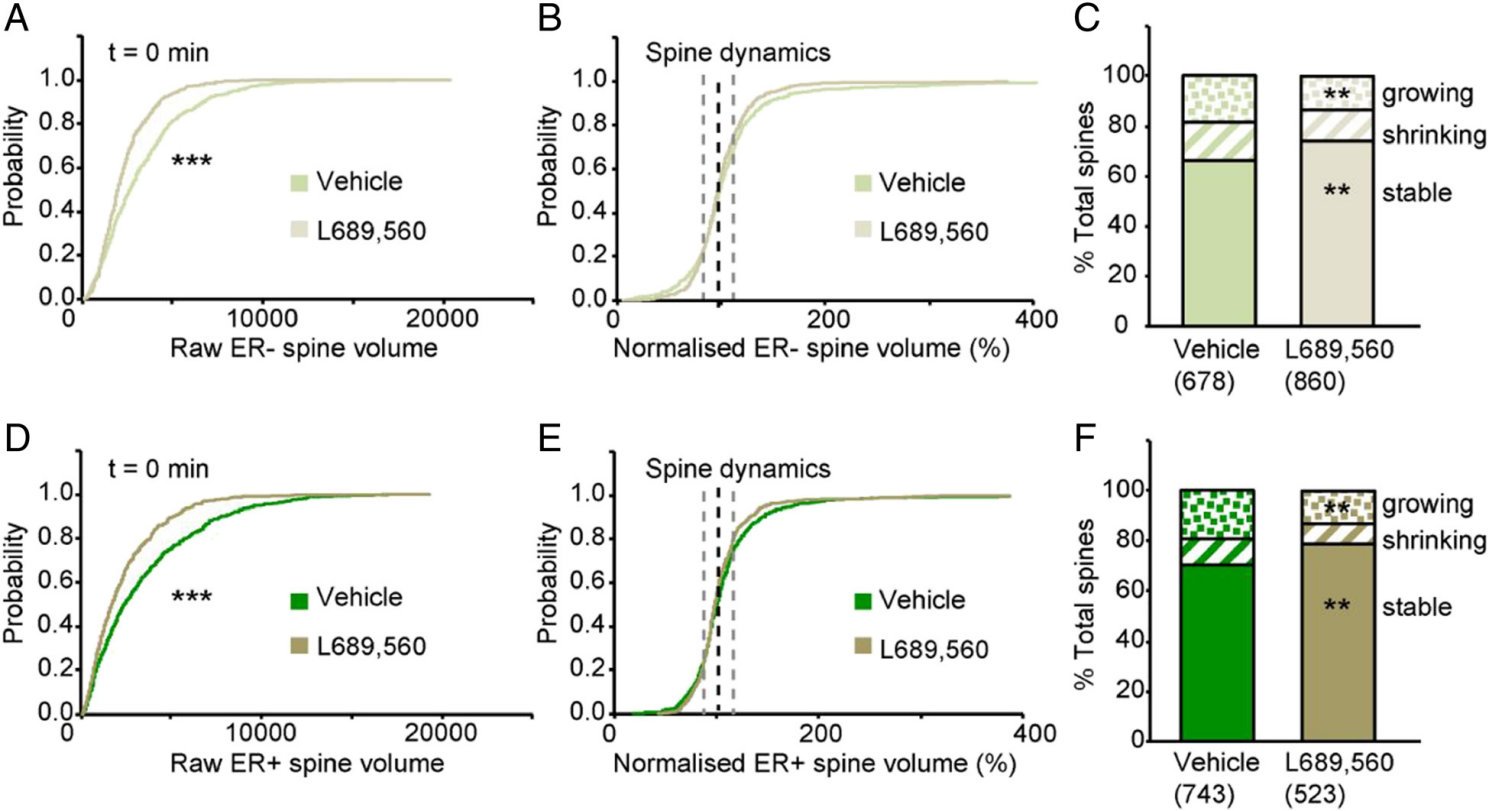
**NMDA receptors activation promotes spine growth and dynamics. (A-C)**. Analysis of ER- spines. **(A)** Cumulative probability plot of spine volume at t = 0 for vehicle and L689,560 groups (K-S test, P < 0.001). **(B)** Cumulative probability plot of normalised spine volumes for vehicle and L689,560 groups. **(C)** Spine distributions for vehicle and L689,560 groups (Z test, P < 0.01 for stable and growing). **(D-F)** Equivalent measures for ER + spines.

Firstly, we analyzed ER- spines. Pre-incubation of the neurons with L689,560 for 10 min resulted in a higher proportion of smaller spines at t = 0 (Figure 3A). Further treatment for 10 min with L689,560 had no additional effects on the spine distribution (data not shown) but reduced spine dynamics, as assessed by comparing the normalised cumulative probability plots for spine volume (Figure 3B). There was an increase in the proportion of “stable” spines (from 66% to 74%) and a corresponding reduction in both the proportion of growing spines (from 18% to 13%) and shrinking spines (from 16% to 13%) (Figure 3C). Therefore, spontaneous synaptic NMDA receptor activity rapidly drives both increases and decreases in spine dynamics.

Next we analysed the ER + spines. The effects of NMDA receptor inhibition were similar to that of ER- spines (Figure 3D-F). Thus, L689,560 reduced overall spine volume and spine dynamics (Figure 3D-E), resulting in an increase in the proportion of “stable spines” (from 70% to 79%), with a concomitant reduction in the proportion of both growing (from 19% to 13%) and shrinking spines (from 11% to 8%) (Figure 3F).

**Figure 4:**
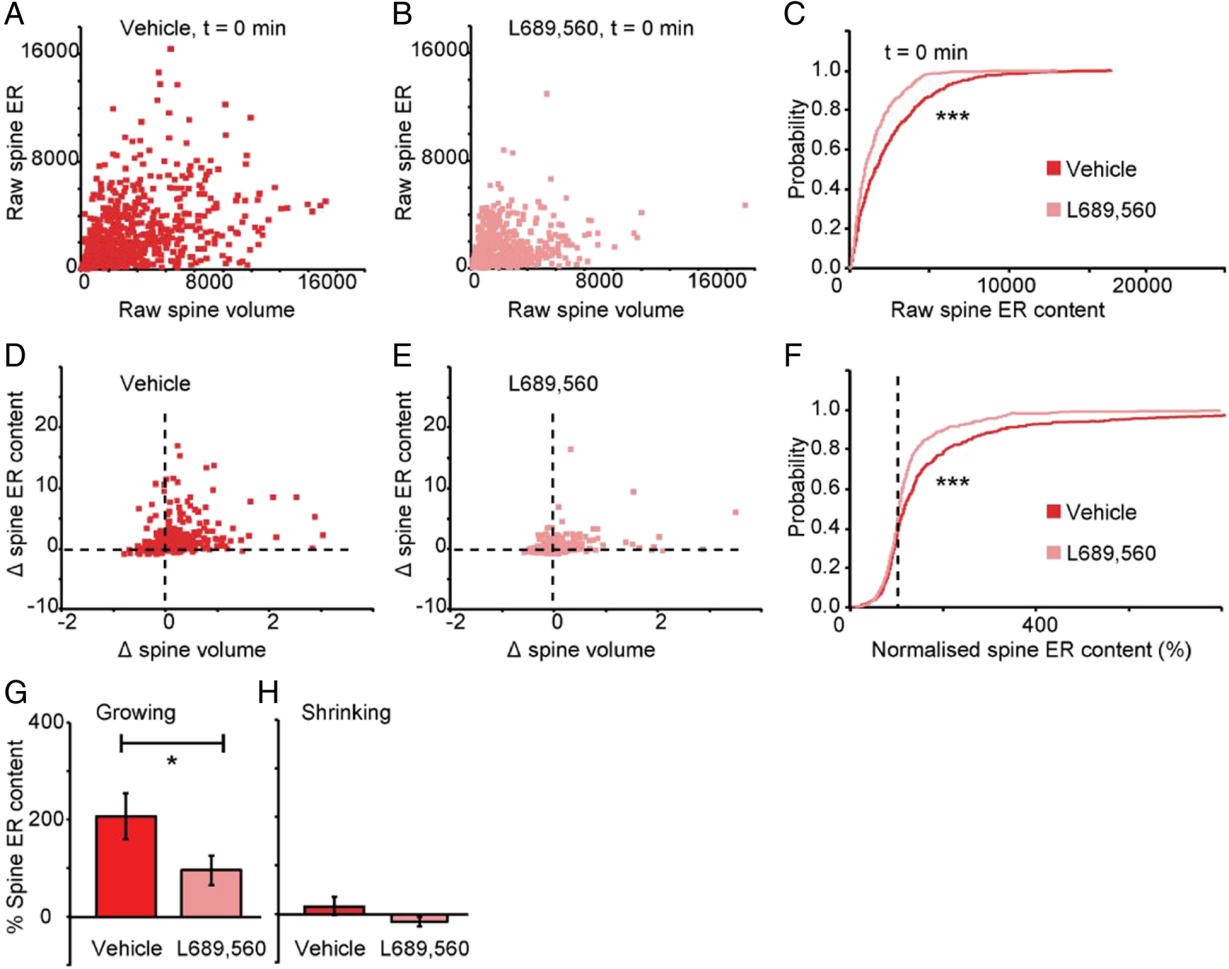
**NMDA receptors activation promotes ER dynamics and expansion. (A)** Scatter plot showing correlation between spine ER content and spine volume at t = 0 min for vehicle. **(B)** Equivalent plot for L689,560. **(C)** Cumulative probability plot of ER volume at t = 0 for vehicle and L689,560 groups (K-S test, P < 0.001). **(D)** Scatter plot for changes in the spine ER content as a function of changes in spine volume over the 10 min period for vehicle (calculated by dividing the difference during the 10 min period by the value at t = 0). **(E)** Equivalent plot for L689,560. **(F)** Cumulative probability plot of normalised ER volume for vehicle and L689,560 groups (K-S test, P < 0.001). **(G)** Changes in ER volume of growing spines for vehicle and L689,560 groups (Mann–Whitney, P < 0.05). **(H)** Changes in ER volume of shrinking spines for vehicle and L689,560 groups.

In terms of ER content of spines, pre-incubation with L689,560 resulted in a decrease in ER volume (as assessed at t = 0). This was apparent in the comparison of the scatter plots of spine ER content versus spine volume (Figure 4A-B) and the cumulative distribution plot of spine ER content (Figure 4C). Furthermore, both the scatter plots (Figure 4D-E) and the cumulative distribution plot of normalised spine ER volume (Figure 4F), calculated during the subsequent 10 min period, shows a reduction in the rate of ER growth. When the growing and shrinking spines were analysed separately, it could be seen that this change was preferentially associated with growing spines (Figure 4G-H). In summary, NMDA receptor activation results in an increase in spine ER content.

### STEP provides a tonic inhibition on spine ER dynamics

**Figure 5:**
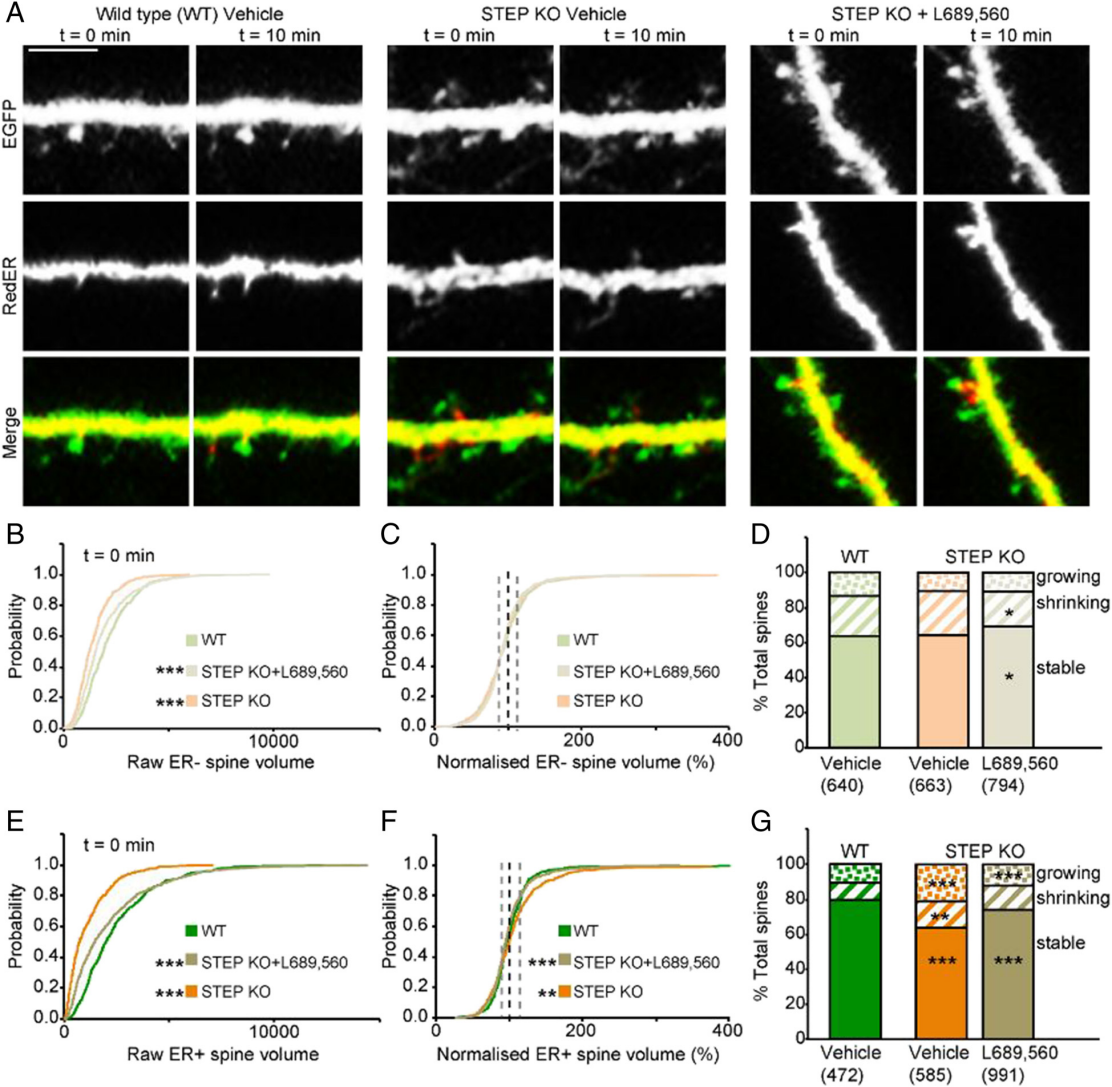
**The regulation of spine dynamics by STEP. (A)** Representative images of the same dendrite at two time points from wild type, STEP KO and STEP KO plus L689,560. Scale bar (applies to all panels): 5 μm. **(B-D)**. Analysis of ER- spines. **(B)** Cumulative probability plot of ER- spine volume at t = 0 for wild type, STEP KO and STEP KO pre-treated for 10 min with L689,560 (K-S test, P < 0.001 between WT and STEP KO and P < 0.001 between STEP KO and STEP KO with L689,560). **(C)** Equivalent plots for normalised spine volumes. **(D)** Spine distributions for the three groups (Z test, P < 0.05 between STEP KO and STEP KO with L689,560 for stable and shrinking). **(E-G)** Equivalent plots for ER + spines. **(E)** Cumulative probability plot of ER + spine volume at t = 0 for wild type, STEP KO and STEP KO with L689,560 (K-S test, P < 0.001 between WT and STEP KO and P < 0.001 between STEP KO and STEP KO with L689,560). **(F)** Equivalent plots for normalised spine volumes (K-S test, P < 0.01 between WT and STEP KO and P < 0.001 between STEP KO and STEP KO with L689,560). **(G)** Spine distributions for the three groups (Z test, WT versus STEP KO or STEP KO versus STEP KO with L689,560).

The effects of the blockade of endogenous NMDA receptor activity can be summarised as a net decrease in spine size, a reduction in spine dynamics and reduced ER expansion in growing spines. So what are the effects of increasing NMDA receptor activity? Activating NMDA receptors exogenously by the addition of NMDA application leads to ER fragmentation [24]. We therefore chose to increase NMDA receptor function by eliminating an endogenous regulator, the protein tyrosine phosphatase STEP [13],[15],[16],[19]. We analysed a total of 1,112 spines on 20 wild type neurons, 1,248 spines on 24 STEP knockout neurons and 1,785 spines on 26 STEP knockouts neurons treated with L689,560 (Figure 5A).

Firstly we analysed ER- spines. Neurons cultured from STEP KO mice displayed a larger proportion of smaller spines compared with neurons cultured from interleaved wild type mice (Figure 5B). Remarkably, pre-incubation of neurons from STEP KO mice with L689,560 for 10 min resulted in a phenotype that was similar to wild type neurons (Figure 5B). A further 10 min treatment with L689,560 produced little additional effect (data not shown). Thus when we investigated the dynamic changes within individual spines, by comparing the normalised cumulative probability plots for spine volume, there was no difference between the STEP KO and wild type groups (Figure 5C-D).

Next, we analysed ER + spines. Again there was a larger proportion of smaller spines in the STEP KO that was reversed by pre-treatment with L689,560 (Figure 5E). However, when we investigated the changes within individual spines over a 10 min period there was a difference between the STEP KO and wild type groups (Figure 5F). Thus, there was a pronounced increase in the proportion of growing spines in the STEP KO (Figure 5F-G). Strikingly, this difference was largely reversed by treatment with L689,560 (Figure 5F-G).

In summary, elimination of STEP resulted in a greater proportion of small spines and this was mainly due to the enhanced activation of NMDA receptors. Additionally, it resulted in an NMDA receptor-dependent increase in the number of growing ER + spines.

**Figure 6:**
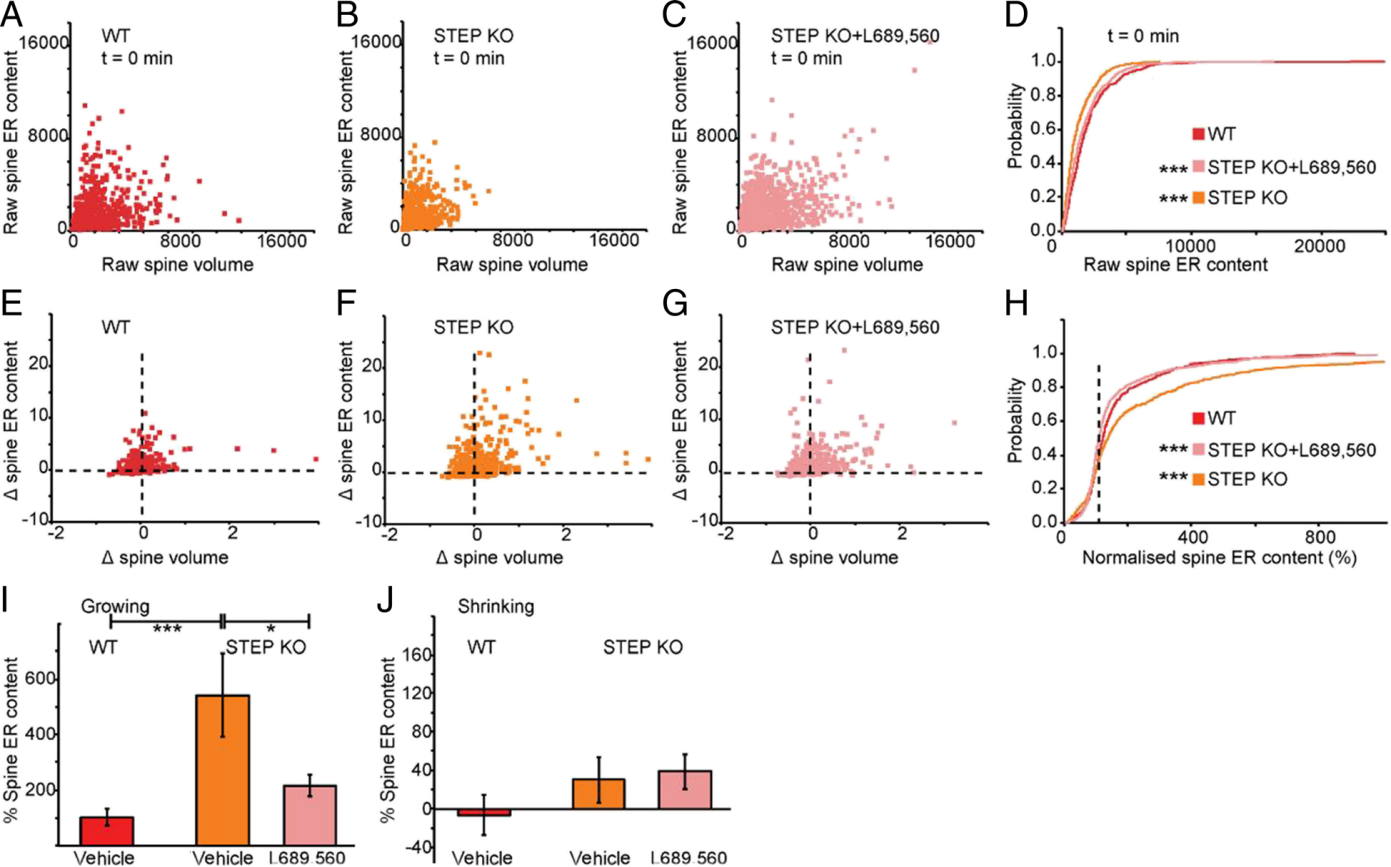
**The regulation of spine ER dynamics by STEP. (A)** Scatter plot showing correlation between spine ER content and spine volume at t = 0 min for WT. Equivalent plot for **(B)** STEP KO and **(C)** STEP KO pre-treated for 10 min with L689,560. **(D)** Cumulative probability plot of ER volume at t = 0 for WT, STEP KO and STEP KO with L689,560 (K-S test, P < 0.001 between WT and STEP KO and P < 0.001 between STEP KO and STEP KO + L689,560). **(E)** Scatter plot for changes in the spine ER content as a function of changes in spine volume over the 10 min period for WT, treated with vehicle. Equivalent plots for **(F)** STEP KO and **(G)** STEP KO with L689,560. **(H)** Cumulative probability plot of normalised spine volumes (K-S test, P < 0.001 between WT and STEP KO or between STEP KO and STEP KO + L689,560). **(I)** Changes in ER volume for the three groups in growing spines (Mann–Whitney, P < 0.001 between WT and STEP KO and P < 0.05 between STEP KO and STEP KO + L689,560). **(J)** Changes in ER volume for the three groups in shrinking spines.

Overall ER volume in STEP KO mice was somewhat smaller than in wild type mice at t = 0, an effect that was reversed by the application of L689,560 (Figure 6A-D). In term of ER dynamics, STEP KO neurons showed a greater rate of ER growth than wild type neurons and this was partially reversed by L689,560 (Figure 6E-H). This was most pronounced in the growing spine subset (Figure 6I). The increase in ER content (at t =10 relative to t = 0) were 103 ± 30%, 542 ± 151%, and 216 ± 37%, for wild type, STEP KO and STEP KO treated with L689,560, respectively (Figure 6I). There was also slight ER expansion in the shrinking spines, but this was unaffected by L689,560 (Figure 6J).

These results suggest that STEP provides a tonic inhibition of ER growth in spines via its regulation of NMDA receptors.

## Discussion

The principal finding of the present study is that the synaptic activation of NMDA receptors leads to very rapid changes in the ER content of dendritic spines. We also found that STEP provides an endogenous negative regulation of this process.

### Morphological changes associated with the synaptic activation of NMDA receptors

Previous studies have shown that the activation of NMDA receptors can influence spine dynamics [7]–[12]. In the present study the extent of this regulation was very pronounced, with dramatic changes being apparent over periods of a few minutes. This presumably reflects our use of dissociated cultures obtained from embryonic tissue that are maintained at 37°C. NMDA receptor activity drove both increases and decreases in spine volume, which may reflect long-term potentiation (LTP) and long-term depression (LTD)-like processes. However, the net effect was an increase in spine growth.

### Rapid alterations in spine ER driven by the activation of NMDA receptors

The present focus was on the ER dynamics that were associated with the spine morphological changes. In this study approximately 50% of the spines analysed contained ER at one or both of the imaging episodes. There was considerable overlap in the morphology of ER + and ER- spines. This contrasts with reports that ER is mainly associated with large mushroom-shaped spines in mature tissue [1],[4]. This may because the spines that we have analysed represent an early developmental stage.

Strikingly, ER was highly dynamic and was, like spine morphology, regulated by NMDA receptor activity. Previous studies have shown that strong NMDA receptor activity, induced by exogenous application of NMDA, leads to ER fragmentation [24]. In the present study we limited our study to the endogenous regulation of NMDA receptors. The most striking observation was the large extent to which acute antagonism of NMDA receptor activity rapidly affected both spine morphology and ER content of spines. This presumably reflects the high level of NMDA receptor activity that is driven by spontaneous synaptic activity in dissociated neuronal preparations. In more intact tissues, such as brain slices, NMDA receptor activity is minimised by the presence of strong GABA-mediated inhibition that limits the synaptic activation of NMDA receptors except during periods of high frequency stimulation [25]. Nevertheless, it is reasonable to suppose that the downstream consequences of NMDA receptor activation, namely changes in spine morphology and spine ER are similar. In other words, the use of a reduced preparation has accentuated a normal physiological mechanism. This has enabled us to more readily investigate some of the regulatory steps involved.

### The regulation of spine ER by STEP

NMDA receptors are dynamically regulated through the opposing activities of serine/threonine or tyrosine kinases and phosphatases within the NMDA receptor complex [26],[27]. In particular, members of the Src-family of tyrosine kinases have been identified as enzymes that enhance NMDA receptor function while the tyrosine phosphatase STEP serves as the tonic brake by opposing the enhancement of Src-family kinase signaling on NMDA receptors [13]–[19],[28]. Blockade of endogenous STEP activity specifically potentiates the NMDA receptor-mediated component of synaptic transmission in CA1 neurons [16].

Our results reveal that STEP also provides a negative regulation of the ability of NMDA receptor activation to promote ER growth in spines. We showed that knockout of STEP protein results in increased ER growth in hippocampal dendritic spines. This suggests that, in addition to its role as a tonic brake on synaptic transmission, STEP regulates ER growth in spines via NMDA receptor dependent pathways. This effect could be mediated exclusively at the level of the NMDA receptor. Thus, by removing STEP there is an enhancement of Fyn/Src-dependent facilitation of NMDA receptor function. The effect of STEP elimination on spine dynamics was only observed in ER + spines. This is consistent with the localisation of STEP with ER membrane [29]. However, we cannot exclude an NMDA receptor-independent effect of STEP contributing to some of the changes in spine and ER dynamics [19].

Since NMDA receptor activation drives net spine growth, it was surprising that in the STEP KO mice there was a greater proportion of smaller spines. This is possibly due to a compensatory reduction in spine size due to the constitutive elimination of an endogenous regulator of the NMDA receptor, which might otherwise lead to excitotoxicity. Another possibility is that the chronic increase in NMDA receptor activity induces LTD, resulting in a smaller spine population. The reduction in spine volume in the STEP KOs was greater in ER + spines compared with ER- spines, which may be due to an influence of Ca^2+^ induced Ca^2+^ release from ER. Interestingly the differences in spine volume between the STEP KOs and wild type mice is caused by continuous NMDA receptor activation as blockade of these receptors leads to a rapid increase in spine volume back to near wild type levels.

### Functional implications for the regulation of spine ER

ER in spines are likely to have multiple functions. For example, smooth ER can serve as both a source and sink of cytoplasmic Ca^2+^ and thereby influence processes such as synaptic plasticity. Indeed, it is established that Ca^2+^ release from intracellular stores results in a large magnification of Ca^2+^ transient that is induced by the synaptic activation of NMDA receptors [30],[31]. How the activation of NMDA receptors leads to ER expansion is not known. One possibility, however, is that the Ca^2+^ release from ER triggers the morphological change. In this way, synaptic NMDA receptor activity, downstream signaling and changes in ER dynamics would be tightly coupled. ER is also important for protein synthesis and protein trafficking, both of which are key components of various forms of synaptic plasticity.

In conclusion, our data suggest that the synaptic activation of NMDA receptors coordinates spine and ER dynamics, which is principally reflected in the rapid expansion of ER in growing spines. The mechanistic link between these processes is not known, but is likely to involve cytoskeletal elements. Clearly, however, STEP provides a brake on the NMDA receptor regulation of ER dynamics.

## Methods

### Animals

Animals were housed under a 12 h light/dark cycle and allowed access to food and water ad libitum. All procedures were carried out in accordance with the Animals (Scientific Procedures) Act 1986.

### Primary hippocampal neuronal cultures and transfection

The disaggregated hippocampi isolated from embryonic day 17 pregnant CD1 (Harlan), wild type C57 (Harlan) or STEP knockout mouse (crossbred for >10 generations in C57) [32] were washed twice in HBSS and transferred to Neurobasal medium supplemented with 2% B-27, 0.5 mM L-glutamine, 1x penicillin/streptomycin (all from Invitrogen) and 25 μM glutamate (Sigma) as described [5]. The cells were seeded at 2×10^5^ cells/dish in 35×100 mm confocal dish (PAA) coated with 10 μg/ml Poly-D-lysine and 5 μg/ml laminin (all from Sigma). At day in vitro (DIV) 5, half of the culture medium was replaced with Neurobasal medium supplemented as above excluding glutamate. At DIV 10, cells were transfected with EGFP-N1 (EGFP; Clontech) and pDsRed2-ER (RedER; Clontech) using Lipofectamine 2000 (Invitrogen). Liposome-containing medium was replaced after 1 h with glutamate-free medium. Experiments were performed on DIV 17–19.

### Genotyping by PCR

Genomic DNA from the respective tail tips or neuronal cultures was isolated using DNeasy tissue kit (Qiagen) and amplified as described [33]. The amplified products were resolved and visualized in 2% agarose gel stained with ethidium bromide.

### Live-cell imaging

Cultures were imaged at 37°C on a Leica SP5 confocal laser scanning microscope attached to an inverted Leica DM I6000 epifluorecence microscope. This system was equipped with a motorised XYZ stage and an environmental chamber (Life Imaging Services) for multi-site imaging while maintaining the temperature at 37°C and CO_2_ at 5%. With the accouto-optical beam splitter (AOBS) module, EGFP fluorescence signal was detected at 498–551 nm and RedER at 572–676 nm. The full-width-at-half-maximal of 0.2 μm laterally and 0.6 μm axially was estimated using 100 nm fluorescent beads as described [34]. On the imaging day, neurobasal culture medium was replaced with HEPES-buffered saline (HBS (mM): NaCl 137, KCl 5, glucose 15, HEPES 25, CaCl_2_ 1.5, MgCl_2_ 1.5; pH adjusted to 7.4 with Tris). The cells were allowed to equilibrate for 10 min in HBS at 37°C before imaging the first time point. Typically, three multipolar spiny neurons per dish were imaged. The x, y coordinates were stored for imaging at different time points. Image stacks at each time points were projected as maximum projections and exported as tagged image files (TIFs). Channels were split in Photoshop (Adobe, San Jose, CA, USA) and mounted as an image stack per neuron in Image J in the following order: time point (TP) 0 min and TP 10 min.

### Data and statistical analysis

For quantification, an image stack for each neuron was merged into a single plane and analysed by a person blinded to the experimental conditions using Volocity 6.0 (Perkin Elmer). Regions of interest (ROIs) were drawn around spines well separated from the dendritic shaft. Estimated spine volume and ER content in spine was measured using integrated pixel intensity as described [35]. Briefly, spine volume was derived from the EGFP-spine ROI after correction against the “adjacent background” (integrated pixel intensity from a defined background ROI proximal to the dendritic shaft). The ER content in each spine was estimated as above from background-subtracted RedER using the same ROI from EGFP-spine measurement. This allowed the RedER signal to be correlated with the respective EGFP-spine volume and compared between time points. Approximately 60 spines from three defined secondary or tertiary dendritic shafts per neuron were analysed and classified into two groups based on whether they contained detectable levels of ER (ER+) at any time points during the imaging period or not (ER-). A spine was considered “stable” if the ratio of integrated pixel intensity was within 25% of its baseline value (i.e. F/F_0_ = ± 25%). The 25% cut off represents approximately 1 standard deviation from the zero value for baseline fluctuation in spine volume. Data are reported as mean ± standard error of the mean (S.E.M). Significance between groups was assessed with two-tailed Mann–Whitney U. A Z-test was used to assess for significance between two proportions. Correlation between changes in spine volume and spine ER content was assessed with Spearman correlation test. Kolmogorov-Smirnov test was used to assess the distributions of the data. P value less than 0.05 was considered significant and shown on the figures as * p < 0.05, ** p < 0.01 and *** p < 0.001. The number of spines analysed is presented in brackets under each histogram.

### Drugs

L689,560 (Tocris) was dissolved in DMSO and frozen in small aliquots at a concentration of at least 1000x the working concentration of 5 μM.

## Competing interests

The authors have declared that no competing interests exist.

## Authors’ contributions

ANN designed and performed the experiments, analysed the data and wrote the first draft of the manuscript. AJD, PJL, NJE and GLC contributed reagents, materials, and analysis tool and helped with the manuscript. All authors have read and approved the final manuscript.
